# Near-UV electroluminescence in unipolar-doped, bipolar-tunneling GaN/AlN heterostructures

**DOI:** 10.1038/lsa.2017.150

**Published:** 2018-02-23

**Authors:** Tyler A Growden, Weidong Zhang, Elliott R Brown, David F Storm, David J Meyer, Paul R Berger

**Affiliations:** 1 Department of Electrical and Computer Engineering, The Ohio State University, Columbus, Ohio 43210, USA; 2 Departments of Physics and Electrical Engineering, Wright State University, Dayton, Ohio 45435, USA; 3 US Naval Research Laboratory, Washington, DC 20375, USA

**Keywords:** AlN, GaN, heterostructure, Near-UV light emission, unipolar, Zener tunneling

## Abstract

Cross-gap light emission is reported in n-type unipolar GaN/AlN double-barrier heterostructure diodes at room temperature. Three different designs were grown on semi-insulating bulk GaN substrates using molecular beam epitaxy (MBE). All samples displayed a single electroluminescent spectral peak at 360 nm with full-width at half-maximum (FWHM) values no greater than 16 nm and an external quantum efficiency (EQE) of ≈0.0074% at 18.8 mA. In contrast to traditional GaN light emitters, p-type doping and p-contacts are completely avoided, and instead, holes are created in the GaN on the emitter side of the tunneling structure by direct interband (that is, Zener) tunneling from the valence band to the conduction band on the collector side. The Zener tunneling is enhanced by the high electric fields (~5 × 10^6^ V cm^−1^) created by the notably large polarization-induced sheet charge at the interfaces between the AlN and GaN.

## Introduction

Since the announcement of the first strong GaN blue-color light-emitting diodes (LEDs) by Nakamura *et al.* in 1991 and 1993^1, 2^, interest in GaN photonics has grown steadily, and commercial applications have expanded to the extent that GaN devices are currently a viable industry. One of the key steps forward by Nakamura *et al.* was the development of a high-quality p-type GaN epitaxial layer using Mg as a dopant. This process allowed the growth of a traditional p-n junction LED with qualities similar to those demonstrated in GaAs since the 1960s. However, the p-type GaN contact remains a problem because it is difficult to grow and has low mobility for uniform carrier injection^3^. This feature becomes a bottleneck in design of GaN-based LEDs, and it is cited as the direct or indirect source of the ‘efficiency droop’ as LEDs are driven towards high brightness applications^4, 5^. In this work, we demonstrate a new pathway with a unipolar n-doped GaN/AlN double-barrier heterostructure light emitter that completely eliminates the need for p-type GaN doping and all of its complications. Instead of injection from a p-contact, holes tunnel into the radiative recombination region, that is, the n-doped GaN emitter, after they are generated by electron Zener tunneling from a valence-band quantum well, which occurs in the GaN spacer at the interface with the AlN barrier on the collector side of the heterostructure. We emphasize that the Zener tunneling is greatly enhanced by the peculiar band bending in the GaN/AlN heterostructure and the resultant small valence-band AlN barriers for the holes in the GaN. This property results from the fact that the internal electric field is both large in magnitude (up to ~5 MV cm^−1^) and opposite in sign between the GaN and the AlN barriers. This claim is justified below in the non-equilibrium Green’s function (NEGF) simulations.

The unique and essential feature of our unipolar-doped GaN light emitter is that strong ‘bipolar tunneling’ can occur in the GaN/AlN heterostructure. Electron current density in the forward direction of the applied bias is augmented by a resonant-tunneling effect, and the hole current density in the backward direction is encouraged by the small AlN hole barriers from the built-in polarization effects between AlN and GaN in their hexagonal crystalline forms. Hence, we refer to this process as unipolar-doped, bipolar-tunneling (UDBT), and to the best of our knowledge, this is the first utilization of such an effect in optoelectronic devices of any sort.

## Materials and methods

### Material growth

The samples were synthesized via plasma-assisted molecular-beam epitaxy (PAMBE) at 860 °C on freestanding Ga-polar semi-insulating GaN substrates grown separately using hydride vapor phase epitaxy (HVPE; Kyma Technologies, Inc.)^6^. The substrates have dislocation densities of approximately 10^6^ cm^−2^. The substrate wafers were cleaned using an aggressive wet chemical etch prior to loading in the ultrahigh vacuum MBE system^7^. Once loaded into the high vacuum, the wafers were de-gassed for 30 min at 600 °C and transferred into the MBE deposition chamber. All samples were grown continuously and without interrupts. Further details on the growth techniques can be found elsewhere^8^.

**Figure 1:**
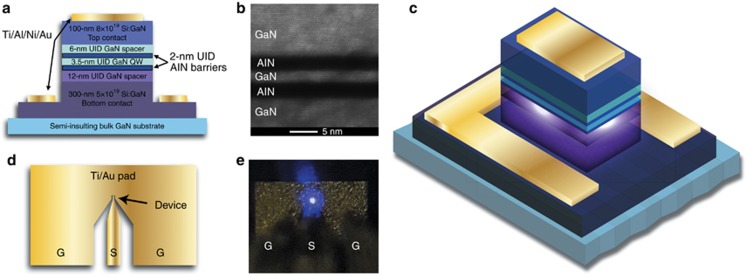
(**a**) Illustration of the baseline device growth stack (Sample A). (**b**) HAADF-STEM image of similarly grown test structure. (**c**) Isometric drawing of the device prior to oxide and pad metal depositions. (**d**) Top–down image of the Ti/Au pad contact. (**e**) Photograph of 7 × 10 μm^2^ GaN RTD structure showing the three DC-coupled electrodes and the RTD mesa device under test (DUT). Strong violet light was observed emitting from the RTD structures under bias, but this was identified as the long-wavelength tail of a much stronger near-UV emission at approximately 360 nm.

Three different unipolar n-doped GaN/AlN resonant tunneling LEDs (RT-LEDs) are presented in this work. The baseline design, Sample A, is displayed in Figure 1a and summarized in Table 1 together with Samples B and C. This material consists of (from the bottom up) a 300-nm-thick GaN:Si bottom contact layer (n-type emitter), a 12-nm unintentionally doped (UID) GaN emitter ‘spacer’ layer, a 2-nm AlN barrier layer, a 3-nm UID GaN quantum well, a second 2-nm AlN barrier, a 6-nm-thick UID GaN collector spacer layer, and a 100-nm-thick GaN:Si top contact layer (n-type collector). Sample B is the same as Sample A but has a 12-nm-thick UID Al_0.2_Ga_0.8_N emitter spacer in place of the GaN emitter spacer. Sample C is the same as Sample A but increases the GaN quantum well thickness to 3.5 nm. To show the high quality of the heteroepitaxial layers, Figure 1b displays a high-angle annular dark-field (HAADF) scanning transmission electron microscopy (STEM) image of the GaN/AlN double-barrier region of a test structure grown in the same way as Samples A-C. The image shows both the abruptness and smoothness of all four heterointerfaces.

### Fabrication

All three samples were fabricated using a six-mask optical-lithography process designed for high-frequency resonant tunneling diodes (RTDs). This process involves top and bottom Ti/Al/Ni/Au contacts, self-aligned mesa definition, device isolation, passivation, and Ti/Au pad contacts. Great care was taken to ensure that high-quality vertical sidewalls were created and passivated. After an aggressive Cl_2_/BCl_3_/Ar inductively coupled plasma reactive ion etch (ICP-RIE), a wet etch was applied to heal the sidewall damage. A 300-nm SiO_2_ layer was deposited using plasma enhanced chemical vapor deposition (PECVD). Details of the growth and processing techniques used in this study are discussed in greater detail in our previous publications on III-nitride RTDs^9^. For electrical bias, the design includes ground-signal-ground (GSG) probe pads, which largely cover the light emitting mesa diode area together with the anode and cathode electrodes (as illustrated by the top contact and pad configuration in Figure 1c and 1d).

### Testing

The initial room temperature DC electrical characterization of the devices was performed with a semiconductor parameter analyzer using standard tungsten probes. For all subsequent light emission studies, the RT-LEDs were electrically biased through a GSG probe. The light spectrum data were measured with a multi-mode fiber-coupled (concave 50-μm slit grating) spectrometer. This spectrometer is capable of UV-VIS-NIR measurements ranging from 200–1080 nm with 2-nm resolution. The fiber was attached to a second micro-positioner next to the GSG and moved directly above the DUT.

The light intensity was measured using a silicon photodiode with a responsivity ≈0.10 AW^−1^ at 360 nm, and the photocurrent was measured with a picoammeter. The testing environment was kept dark under notably low background illumination such that both *L*–*V* and *L*–*I* curves could be measured with high precision. The photodiode was attached to a rotationally adjustable arm that allowed for measurements at different angles with respect to the DUT. Measurements for the EQE were taken at polar angles in the range of 15–90°.

### Modeling and calculations

Computer simulations were performed using Silvaco’s NEGF formalism in Atlas (with material parameters listed in the Supplementary Information)^10^. This model allows for a self-consistent solution between the Poisson and NEGF equations using an effective mass Hamiltonian. The device is broken into multiple regions of collector, emitter, active non-equilibrium, and reservoirs. The reservoirs (including the spacer) immediately surround the active region and stop at the contacts, which are flatband type. The contacts and the reservoir are both considered to exist in thermodynamic equilibrium, but the occupation factor varies based on the quasi-Fermi level on their respective sides. The active region is considered non-equilibrium. The Green’s functions are calculated for the active region and the two reservoirs. However, the charge for the contacts is calculated with semi-classical techniques. The 1D effective mass Hamiltonian is discretized in real space via a finite difference method.

Alternatively, we have also developed analytical models of the electron currents using standard formulations for comparison. First, we use the inelastic form of the Breit–Wigner transmission probability through a single quasi-bound level in the presence of scattering and integrate it over the Fermi-sea on the emitter side using the standard Tsu-Esaki integral of quantum transport theory^11^. We add to this an electron ‘leakage’ current term to represent a combination of (i) inelastic tunneling at longitudinal energies well away from the quasi-bound level and (ii) thermionic emission over the top of the barriers. The leakage term has the form of the Shockley Equation, *I*_L_=*I*_0_[exp(*α*V/k_B_T) −1], where *I*_0_ and *α* are constants determined by curve-fitting to the experimental data. A **k**·**p** approach is used to evaluate the hole current density using a WKB approximation for the tunneling integral^12, 13^. The potential profiles for the accumulation in the emitter and the depletion in the collector were calculated using the method given in Ref. 14.

## Results and discussion

**Figure 2:**
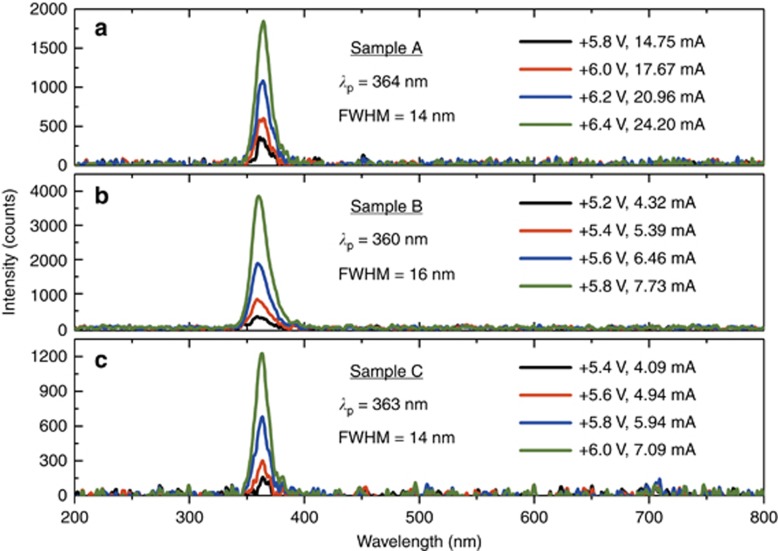
Measured light spectrum emitted from representative 7 × 10 μm^2^ devices as a function of voltage bias for (**a**) Sample A, (**b**) Sample B and (**c**) Sample C. The associated device current is also displayed. All measurements were conducted at room temperature.

**Figure 3:**
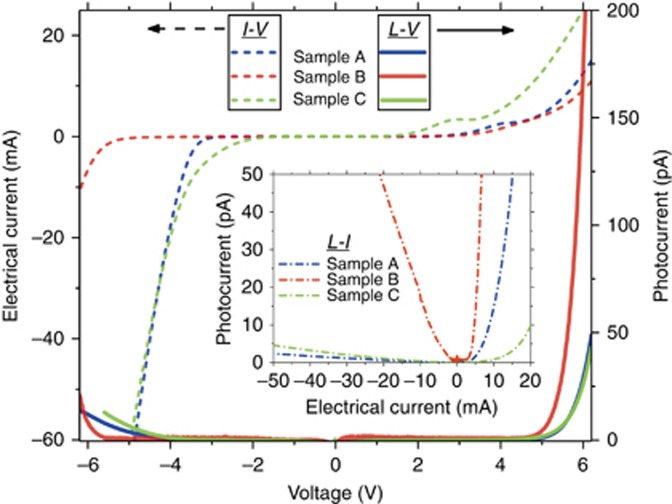
DC *I*–*V* and *L*–*V* curves from all three samples during positive voltage bias and negative voltage bias. The inset displays the *L*–*I* curves for all three samples.

Light emission was initially observed by eye through the probe-station microscope when the RTDs were biased beyond ~5 V. Strong violet light was observed coming from the mesa periphery, as displayed in Figure 1e, with fully repeatable negative differential resistance (NDR) at room temperature (shown later in Figure 3). The light emission was sufficiently bright that it could easily be measured by a commercial grating spectrometer coupled to the device through a bundled fiber probe placed in close proximity. The emission spectra of all three samples (Figure 2a–2c) exhibited a dominant peak centered near 360 nm that increases in intensity with increasing bias voltage and has full-width at half maximum (FWHM) values of 14 nm for Sample A, 16 nm for Sample B and 14 nm for Sample C. While under positive voltage bias, the FWHM remains≤16 nm, even at the highest applied bias levels, with no significant spectral broadening. The 360-nm emission is attributed to cross-gap transitions because the wavelength corresponds closely to the 3.44 eV bandgap of GaN at room temperature^15^. With increasing voltage bias, the emitted light remains quite optically pure in all three samples without any significant sub-bandgap emission, as reported in many other GaN light-emission results. However, the devices studied in this work often failed in the form of a short circuit as the bias was raised above a critical breakdown voltage (for example, ≈7 V).

The room temperature current–voltage (*I*–*V*), light intensity vs voltage (*L*–*V*), and light intensity vs current (*L*–*I*) characteristics for all three samples are displayed in Figure 3. Although both the *L*–*V* and *L*–*I* curves exhibit a threshold effect, the *L*–*I* curves are distinct between the samples, whereas the *L*–*V* curves display a common threshold (~4.7 V). Above the threshold, the *L*–*V* curve displays an exponential increase of light emission vs bias voltage.

**Figure 4:**
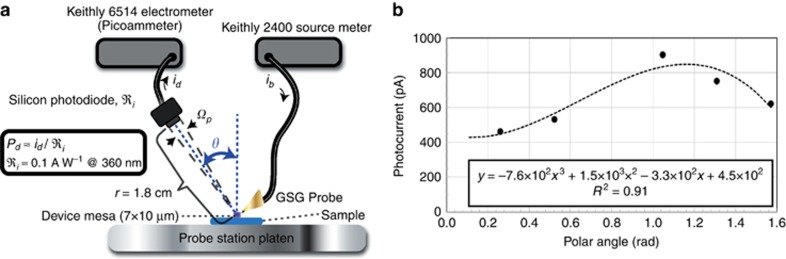
(**a**) Experimental set-up used to measure the emitted power at various angles *θ* from the polar axis and into a solid-angle *Ω_p_* defined by the silicon photodiode area and range *r* from the GaN unipolar-doped light emitter. (**b**) Photocurrent vs elevational angle for Sample B obtained with the set-up in **a**. The data points are shown as solid circles, and the cubic-polynomial curve-fit is shown as a dashed line.

The far-field intensity of the RT-LEDs was measured, despite the shadowing of the GSG probe pads atop the DUT. The far field was found to be significantly dependent on the elevational angle *θ* in Figure 4a but relatively independent of the azimuthal angle *φ*. This observation is consistent with symmetry considerations given that the radiating structure is a mesa with exposed sidewalls around the periphery. Optical measurements at five elevational angles for Sample B are shown in Figure 4b, all at a range of 1.8 cm from the mesa. The resulting data points were fit with a cubic polynomial, and the best fit was *I*(*θ*)=−7.6 × 10^2^*θ*^3^+1.5 × 10^3^*θ*^2^−3.3 × 10^2^*θ*+450. The total power was estimated into the upper hemisphere by a rectangle approximation 
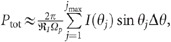
 where ℜ_*I*_≈0.1 AW^−1^ is the current responsivity of the photodiode at 360 nm, and *Ω_p_* is the solid angle subtended by the photodiode with respect to an origin defined by the emitting diode 1.8-cm away, such that *Ω_p_*≈0.010 str. Setting Δ*θ*=1.0° (0.017 rad), we find *P*_tot_=4.7 × 10^−6^ W. The EQE, *η*_ext_^16^, was calculated with additional parameters *I*_*B*_=18.8 mA and *hν*=3.4 eV and led to the lower limit estimation of *η*_ext_≈0.0074%.

This *η*_ext_ value is well below the state-of-the-art values of ~50% for optimized bipolar-doped (p-n) GaN LEDs^17^. However, we emphasize that this value is conservative, considering emission into only the upper hemisphere and ignoring internal loss mechanisms such as total internal reflection. In addition, these devices are not designed to balance electron and hole currents such that the electron-hole radiative recombination reported is currently hole-limited. Even so, to the best of our knowledge, this value is higher than the values reported for any other unipolar-doped GaN emitter to date, such as the 10^−6^% UV-value reported in Zimmler *et al*^18^.

**Figure 5:**
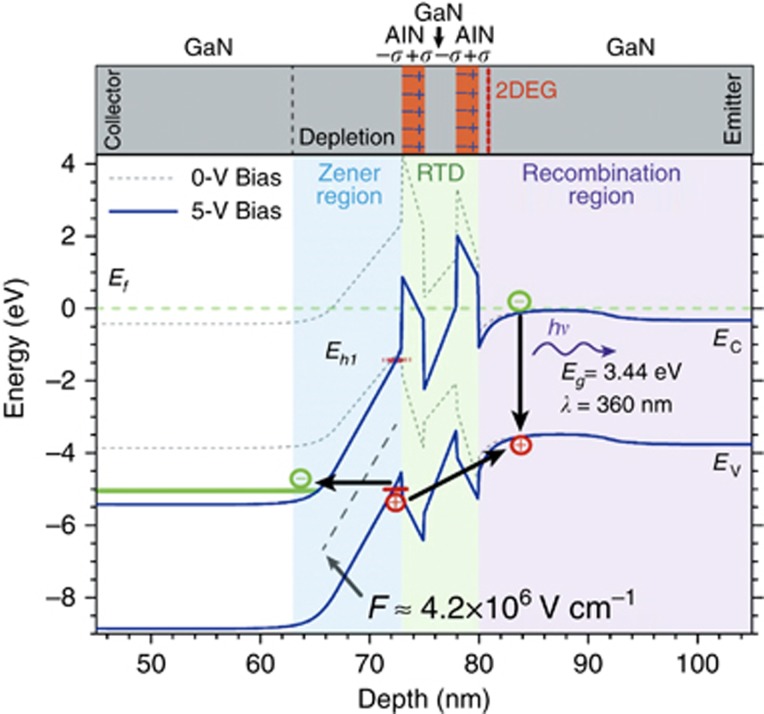
Band diagram of the GaN/AlN heterostructure generated using a NEGF simulation. The holes are generated in the Zener region and subsequently tunnel through the RTD region into the emitter spacer where they recombine. The lack of observable emission from transitions between the bound conduction and valence band states within the quantum well is attributed to the quantum-confined Stark effect, resulting in a small wave function overlap.

Initial NEGF modeling indicates that the holes necessary for the observed cross-gap emission are created by Zener tunneling across the UID GaN collector spacer (Figure 5). A large internal electric field is present as a consequence of polarization-induced charge density caused by two mechanisms: one from piezoelectric polarization because of the abrupt lattice mismatch between AlN (4.982 Å) and GaN (5.185 Å) and the other from the discontinuity of spontaneous polarization between AlN (−0.081 C m^−2^) and GaN (−0.029 C m^−2^)^19, 20, 21, 22, 23, 24, 25^. The induced surface charge density might reach levels of *σ*~5.5 × 10^13^ cm^−2^, which leads to fields approaching 10 MV cm^−1^ in the AlN and at its interface with the GaN layers^20^. These enormous polarization-induced electric fields present in III-nitride heterostructures have been recently confirmed by direct measurement with nano-beam electron diffraction^26^. The induced field creates a depletion region within the UID collector spacer and an accumulation region in the UID emitter spacer. Under the external voltage bias, the field increases further, which makes Zener interband tunneling possible even though the potential barrier (cross-bandgap GaN) is ~3.44 eV. For perspective, if the internal field is *F*=2 MV cm^−1^, the interband hole generation density is estimated to be ~0.66 cm^−3^ s^−1^ with Kane’s model^12^, whereas when it increases to *F*=5 MV cm^−1^, the hole density rate increases to ~3.1 × 10^20^ cm^−3^ s^−^^1^.

**Figure 6:**
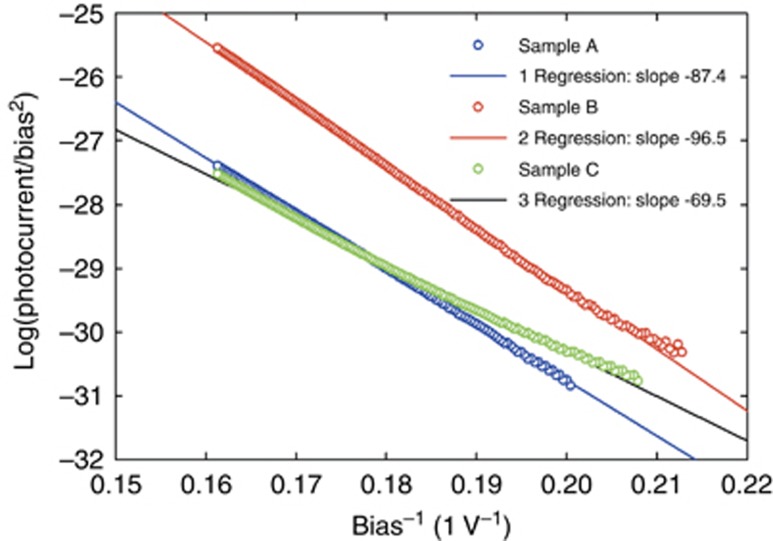
Forward bias fittings of emission vs bias voltage with Kane’s Zener tunneling model^12^.

Once generated, the holes can migrate by tunneling (possibly by Auger recombination as well) to the emitter side of the structure where electron-hole recombination occurs. For small bias, estimations with a Bardeen Transfer Hamiltonian method indicate the hole transmission through the double-barrier structure is smaller than the electron transmission due to the larger light-hole mass (*m*_lz_≈1.1 vs *m*_e_≈0.2 *m*_0_; Supplementary Information), despite a smaller valence band offset barrier (Δ*E*_v_GaN/AlN_≈0.7 eV vs ΔE_c_GaN/AlN_≈2.0 eV Ref. 27). However, the hole transmission increases considerably because the hole quasi-bound level moves downward as the internal field increases (Figure 5). This observation is essential to the ‘bipolar tunneling’ effect of this letter. Fitting of the experimental photocurrent at both bias polarities was conducted, and the results agree well with Kane’s model, thus supporting interband Zener tunneling as the primary source of hole generation (Figure 6). Additionally, previous researchers have reported similar Zener tunneling effects through a thin AlN layer sandwiched between p-type and n-type GaN layers^28, 29, 30, 31, 32^.

Although additional research is necessary, we attribute the occurrence of the new tunneling effect, the bright near-UV emission, and the robustness of the demonstrated devices to the quality of the GaN/AlN heterointerfaces. This observation is supported by material evidence through the ultra-smooth HAADF images in Figure 1b, by electrical evidence through the stability of the *I*–*V* curves and the NDR region (Figure 3), and by photonic evidence through the spectral purity of the emission and the lack of sub-bandgap emission.

To estimate the internal quantum efficiency (IQE), we must first determine the injection efficiency (IE) and the light extraction efficiency (LEE). However, given the uniqueness of the device layout and hole injection, certain assumptions must be made. Because light emission from these devices is hole limited, we can estimate the IE from the ratio between the measured electron and calculated hole current densities (*J*_p_/*J*_n_). Applying this methodology to Sample B results in an IE value of ~1.0%. As mentioned earlier, the current RTD/LED structure was designed for stable NDR at room temperature^8, 9^ and is therefore non-optimal as an LED. The top ‘mesa’ surface is largely covered by a thick metal (>400 nm), resulting in approximately 40%–60% of the surface area emitting light (illustrated in Figure 1c). Additionally, due to a large refractive index difference between GaN (*n*=2.6 at 360 nm) and air (*n*=1.0), the maximum emission efficiency dictated by the narrow escape cone (22.6°) is 3.8%. A concatenation of these effects led us to an LEE estimation of ~1.5%–2.3%. Subsequently, combining the measured EQE for Sample B and the estimated IE and LEE values, we approximate the IQE to fall within a range of ~30%–50% (IQE=EQE/(IE × LEE)). This range seems quite reasonable because the IQE generally reflects the quality of the crystal and these devices were grown on low dislocation bulk GaN and are also functional RTDs^8, 9^, which is indicative of excellent epitaxy.

Increasing the EQE will require substantial improvement in both the IE and LEE, even if deleterious to the RTD performance. The LEE shortcomings can be addressed by simply applying the traditional LED design techniques such as minimal contact coverage, surface roughening^33^, or micro-lens arrays^34^. However, the limiting factor is the notable poor IE, which in the state-of-the-art LED technology is generally thought to approach 100%. Improvement would involve a more evenly balanced electron and hole current ratio.

**Figure 7:**
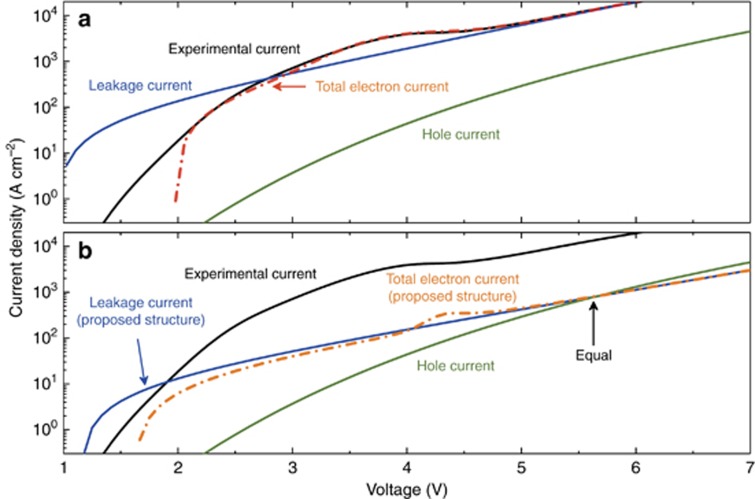
(**a**) Fitting of the experimental electron current of Sample A with the analytic model. (**b**) Proposed approach for balancing electron and hole current densities by reducing the n-type doping concentration on the emitter side. Total electron current is the summation of the leakage current and the resonant tunneling current.

To balance the electron and hole current, we investigated the separate current mechanisms of Sample A using the previously discussed modeling techniques. Figure 7a compares the experimental *J–V* curve (current of Sample A in Figure 3 divided by the device area) against our electron and hole current models. The combination of resonant tunneling and leakage current of the electrons offers a good fit to the experimental *J*–*V* clearly showing the NDR while the hole current is much smaller in comparison. Above ~5.0 V, near where the experimentally measured device displays a threshold in near-UV light emission, the hole current density begins to take off as well. Further investigation shows that the simplest way to shrink the electron-hole difference is to reduce the electron current density while holding the hole density nearly constant. A reduction in the Fermi energy (*E*_F_) on the emitter side does exactly this, and the electron resonant-tunneling and leakage mechanisms both fall monotonically, whereas the Zener-tunneling of holes have practically no dependence on *E*_F_ at all. The n-type doping concentration outside the spacer layer on the emitter side determines *E*_F_, and for the existing structure with *N*_D_=5 × 10^19^ cm^−3^, *E*_F_=0.25 eV using the conduction-band parameter of GaN, and *m*_e_=0.20 *m*_0_. A reduction of *N*_D_ to 5 × 10^18^ cm^−3^ would drop *E*_F_ to 0.05 eV, and the resulting model *J*–*V* curves are plotted in Figure 7b. The electron current drops dramatically but not the hole current such that the two currents are equal at ~5.7 V bias. This simple scaling in an easily controllable material growth parameter should significantly improve the balance between electrons and holes, thereby greatly enhancing the IE.

## Conclusions

Cross-gap GaN emission has been discovered in unipolar n-doped GaN/AlN double-barrier heterostructures. Because these devices were not purposely designed as LEDs, the light-emission efficiency is far from optimized. Upon subsequent optimization of the unipolar LEDs, the electron and hole current injection could be balanced, leading to commensurate external quantum efficiencies of state-of-the-art GaN-based LEDs but without the added steps and complications required by p-type doping.

## Figures and Tables

**Table 1:** Device structures and parameters

Sample	A	B	C
Emitter contact	300-nm GaN 5E19 cm^−3^	300-nm GaN 5E19 cm^−3^	300-nm GaN 5E19 cm^−3^
Emitter spacer	12-nm UID GaN	Digital AlGaN alloy (2.5-Å AlN alternating with 10-Å GaN)	12-nm UID GaN
Barriers	2-nm UID AlN	2-nm UID AlN	2-nm UID AlN
Quantum well	3-nm UID GaN	3-nm UID GaN	3.5-nm UID GaN
Collector spacer	6-nm UID GaN	6-nm UID GaN	6-nm UID GaN
Collector contact	100-nm GaN 8E19 cm^−3^	100-nm GaN 8E19 cm^−3^	100-nm GaN 8E19 cm^−3^
