# Supplementary material for: Near-UV electroluminescence in unipolar-doped, bipolar-tunneling GaN/AlN heterostructures
Source: Light Sci Appl. 2018 Feb 23;7:17150–. doi: 10.1038/lsa.2017.150 (PMC6060061; doi:10.1038/lsa.2017.150)
Supplement: Supplementary Information [file lsa2017150x1.docx]

**Near-UV Electroluminescence in Unipolar-Doped, Bipolar-Tunneling**

**GaN/AlN Heterostructures**

Tyler A. Growden^1^, Weidong Zhang^2^, Elliott R. Brown^2^, David F. Storm^3^, David J. Meyer^3^, and Paul R. Berger^1^*

^1^ Department of Electrical and Computer Engineering, The Ohio State University, Columbus, OH 43210, USA

^2^ Departments of Physics and Electrical Engineering Wright State University, Dayton, OH 45435, USA

^3^ U.S. Naval Research Laboratory, Washington, DC 20375, USA

*Correspondence: pberger@ieee.org

1. **Material Parameters**

**Polarization:**

GaN AlN

Lattice constant *a_0_* (Å): 3.189 3.112

Spont polarization *P_sp_* (Cm^-2^): -0.034 -0.09

Piezoelectric const.(z) *e_33_* (Cm^-2^): 0.67 1.5

Piezoelectric const.(x,y) *e_31_* (Cm^-2^): -0.34 -0.53

Elastic constant *C_33_* (GPa): 392 382

Elastic constant *C_13_* (GPa): 100 127

Region Spontaneous polarization Piezoelectric polarization Total polarization

Number sheet charge (cm^-2^) sheet charge (cm^-2^) sheet charge (cm^-2^)

---------------------------------------------------------------------------------------------------------------

1 (GaN) 2.122e+13 0.000e+00 2.122e+13

2 (AlN) 5.617e+13 3.177e+13 8.795e+13

3 (GaN) 2.122e+13 0.000e+00 2.122e+13

4 (AlN) 5.617e+13 3.177e+13 8.795e+13

5 (GaN) 2.122e+13 0.000e+00 2.122e+13

**Effective mass:**

GaN AlN

Electron effective mass (z) *m_ez_*: 0.2 0.33

Electron effective mass (t) *m_et_*: 0.18 0.25

Hole effective mass parameter *A1*: -7.24 -3.95

Hole effective mass parameter *A2*: -0.51 -0.27

Hole effective mass parameter *A3*: 6.73 3.68

Hole effective mass parameter *A4*: -3.36 -1.84

Hole effective mass parameter *A5*: -3.35 -1.92

Hole effective mass parameter *A6*: -4.72 -2.91

*z* = axial

*t* = transverse

*m_hhz_ = -m*_0_(*A1* + *A3)^-1^*

*m_hht_ = -m*_0_(*A1* + *A3)^-1^*

*m_lhz_ = -m*_0_[*A1* + $(\frac{E_{lh}^{0}- \lambda_{\varepsilon}}{E_{lh}^{0}- E_{ch}^{0}})$*A3*]*^-1^*

*m_lht_ = -m*_0_[*A2* + $(\frac{E_{lh}^{0}- \lambda_{\varepsilon}}{E_{lh}^{0}- E_{ch}^{0}})$*A4*]*^-1^*

*m_chz_ = -m*_0_[*A1* + $(\frac{E_{ch}^{0}- \lambda_{\varepsilon}}{E_{ch}^{0}- E_{lh}^{0}})$*A3*]*^-1^*

*m_cht_ = -m*_0_[*A2* + $(\frac{E_{ch}^{0}- \lambda_{\varepsilon}}{E_{ch}^{0}- E_{lh}^{0}})$*A4*]*^-1^*

**Regional material parameters:**

Region 1 2 3 4 5

Material GaN AlN GaN AlN GaN

Band parameters

Epsilon 8.9 8.5 8.9 8.5 8.9

E_g_ (eV) 3.43 6.14 3.43 6.14 3.43

Chi (eV) 4.31 2.42 4.31 2.42 4.31

N_c_ (cm^-3^) 2.24e+18 4.42e+18 2.24e+18 4.42e+18 2.24e+18

N_v_ (cm^-3^) 2.51e+19 6.76e+18 2.51e+19 6.76e+18 2.51e+19

N_i_ (cm^-3^) 1.06e-10 1.51e-33 1.06e-10 1.51e-33 1.06e-10
